# Regulatory Roles of MicroRNAs in Diabetes

**DOI:** 10.3390/ijms17101729

**Published:** 2016-10-17

**Authors:** Juan Feng, Wanli Xing, Lan Xie

**Affiliations:** 1 Medical Systems Biology Research Center, School of Medicine, Tsinghua University, Beijing 100084, China; fengjuansdu@126.com; 2 National Engineering Research Center for Beijing Biochip Technology, Beijing 102206, China

**Keywords:** miRNAs, diabetes, β-cell function, insulin resistance, circulating miRNAs

## Abstract

MicroRNAs (miRNAs), a class of endogenous small noncoding RNAs in eukaryotes, have been recognized as significant regulators of gene expression through post-transcriptional mechanisms. To date, >2000 miRNAs have been identified in the human genome, and they orchestrate a variety of biological and pathological processes. Disruption of miRNA levels correlates with many diseases, including diabetes mellitus, a complex multifactorial metabolic disorder affecting >400 million people worldwide. miRNAs are involved in the pathogenesis of diabetes mellitus by affecting pancreatic β-cell functions, insulin resistance, or both. In this review, we summarize the investigations of the regulatory roles of important miRNAs in diabetes, as well as the potential of circulating miRNAs as diagnostic markers for diabetes mellitus.

## 1. MicroRNAs (miRNAs) and Diabetes

Diabetes mellitus is a progressive metabolic disease that is characterized by high blood sugar and is a great threat to human health. According to the World Health Organization, there are currently >400 million people suffering from diabetes worldwide, and that number will reach 552 million by 2030 [1]. Diabetes was first reported by the ancient Egyptians nearly 3000 years ago [2]. In 1936, the distinction between type 1 diabetes (T1DM) and type 2 diabetes (T2DM) was clearly defined [3]. Among patients diagnosed with diabetes, T1DM accounts for 5%–10% with the other ~90% having T2DM. T1DM is a form of diabetes mellitus in which not enough insulin is produced by islet cells in the pancreas and subsequently results in high blood sugar levels. However, the exact cause of T1DM is still unknown. In most cases, it is an autoimmune disease, a condition in which the immune system mistakenly attacks the insulin-producing cells in the pancreas. T1DM is mostly diagnosed in children, adolescents, or young adults [3]. T2DM is featured with high blood sugar, insulin resistance (IR), and relative deficiency of insulin. The occurrence of T2DM results from a combination of genetic, environmental, and behavioral risk factors [4]. Different from T1DM, T2DM is often adult onset.

MicroRNAs (miRNAs) are short, non-coding RNAs with a size of ~22 nt [5]. In most cases, miRNA acts as negative regulators at the post-transcriptional level by inhibiting mRNA translation or degrading the mRNA by complementary binding to its 3′-untranslated region (3′-UTR) via the seed sequence region at the 5′ end of the miRNA. miRNAs are estimated to affect approximately 30% of protein coding genes [6]. An aberrant expression of miRNAs interferes with both physiological and pathological processes.

Since the discovery of miRNAs, an increasing number of them have been found involved in diabetes mellitus pathogenesis [7]. Dysregulation of miRNA can lead to profound impairment of glucose metabolism [8]. miRNA expression profiles of various tissues (e.g., pancreas, adipose tissue, and liver) from T2DM patients or hyperglycemia animal models have been established in recent years and make it easier to uncover novel miRNA regulators in diabetes.

## 2. miRNAs and Pancreatic β-Cells

Pancreatic β-cells play a central role in glucose homeostasis through secretion of insulin [9]. Important miRNAs associated with pancreatic β-cell dysfunction are summarized in Table 1. Generally, they affect β-cells through the regulation of cell survival and apoptosis, proliferation, differentiation, or function, especially insulin secretion.

To regulate β-cell survival and apoptosis, miRNAs usually function by targeting cell apoptosis-related genes, such as the pro-apoptotic gene *Bax* (full names of all gene symbols are summarized in Appendix Table A1) and the anti-apoptotic gene *Bcl-2* [10,11]. For example, Belgard et al. show that overexpression of the *miR-200* family in mice is sufficient to induce β-cell apoptosis and lethal T2DM through indirect activation of the pro-apoptotic genes *Trp53* and *Bax* [12], while elevated *miR-34a* promotes MIN6 cell apoptosis by directly binding to the 3’-UTR of the anti-apoptotic gene *Bcl-2* [13].

In β-cell proliferation, some miRNAs play positive roles, while other miRNAs exhibit negative effects. One of the most important miRNA regulators is *miR-375*, which is highly expressed in both human and mouse pancreatic β-cells and is indispensable in maintaining normal pancreatic β-cell mass. Mice lacking *miR-375* (375KO) display decreased pancreatic β-cell mass as a result of impaired proliferation, and further analysis demonstrates that *miR-375* works by targeting a number of growth-inhibiting genes and is required for β-cell proliferation [14]. For example, *Cadm1*, as the direct target gene of *miR-375*, negatively regulates the G1/S transition and represses cell growth in various cancer cells lines [15]. Similar protective effects have already been reported for *miR-181a* because the reduction of *miR-181a* in the islets of young rats also impairs β-cell proliferation [16]. Besides, *miR-17* could promote β-cell proliferation through directly targeting *Menin*, which plays a crucial role in the regulation of gene transcription, cell cycle and apoptosis, in several mouse models and MIN-6 cells [17]. Consistently, it is reported that *miR-17/92*, a miRNA cluster expressed in multiple tissues, also promotes pancreatic β-cell proliferation and adaption under stress conditions, further confirming the positive role of *miR-17* in β-cell proliferation [18]. Other pancreatic β-cell proliferation regulators include *miR-24*, which is highly expressed in pancreatic β-cells, further upregulated in the islets of genetic fatty db/db mice, and inhibits β-cell proliferation and insulin secretion by binding to two maturity-onset diabetes of the young genes, *Hnf1α* and *Neurod1*. Meanwhile, the expression of *miR-24* increased from 2.0- to 3.5-fold in 8- and 12-week-old db/db mice, showing an increase with the aging of db/db mice [19]. Further, *miR-29a* suppresses cell proliferation in INS-1E cells, leading to decreased glucose-stimulated insulin secretion of β-cells [20].

In the field of β-cell differentiation, the capacity of human pluripotent stem cells (hPSCs) to differentiate into insulin-producing cells (IPCs) makes them a promising in vitro model to study the regulatory roles of miRNAs in β-cell differentiation. Accordingly, the differentiation of hPSCs in vitro displays four stages: formation of definitive endoderm (0–4 days), induction of pancreatic progenitor cells (PPCs) (5–9 days), expansion of PPCs (10–15 days), and formation of IPCs (16–25 days). Based on this model, specific miRNAs have been proposed to modulate the process of directing hPSC differentiation toward islet-like cell clusters. Dynamic expression of miRNAs during the differentiation of human embryonic stem cells into islet-like cell clusters has been quantified, and four islet-specific miRNAs (*miR-7*, *miR-375*, *miR-34a*, and *miR-146a*) exhibit distinct expression patterns during this process. Among them, *miR-375* and *miR-7* increase from day 4, peak on day 8, and then decline until the end of differentiation. In contrast, *miR-146a* declines throughout the differentiation process, and *miR-34a* expression is reduced initially, followed by restoration on day 21. The following analysis reveals that *miR-375* directly targets *HNF1β*, and overexpression of *miR-375* downregulates the protein level of HNF1β, while *miR-7* directly targets *PAX6*, and overexpression of *miR-7* decreases the expression level of PAX6 [21]. Later studies have also proven that *miR-7* and *miR-375* are essential for pancreatic β-cell differentiation and development [22], and in vitro forced expression of *miR-7* or *miR-375* helps to differentiate hPSCs into IPCs [23,24]. In addition, a number of other miRNAs, including *miR-30d*, *let-7e*, *miR-21*, *miR-9*, and *miR-376*, are also implicated in human pancreatic islet differentiation and development [25,26,27].

Aside from the above, many other miRNAs are involved in pancreatic function, especially insulin secretion (e.g., *miR-375*, -184, *-33*, *-187*, *-29a*, and *-30a*) [28,29,30,31,32,33,34,35,36,37]. For example, although *miR-29* is mentioned above in regard to its role in regulating β-cell proliferation, it has also been shown in multiple reports to negatively regulate insulin secretion by directly targeting *Stx-1a*, a t-SNARE protein involved in insulin exocytosis [31], and *Mct1*, which may affect insulin secretion [32]. *miR-124a* is increased in type 2 diabetic human pancreatic islets and negatively regulates insulin secretion by directly targeting the GTPase *Rab27a* [33] and *Foxa2* [34], which contribute to β-cell dysfunction in T2DM. Taken together, miRNAs and their target genes constitute a complex regulatory network in pancreatic β-cell functions, and the dysregulation of miRNAs may lead to diabetes mellitus.

## 3. miRNAs and Insulin Resistance (IR)

**Figure 1:**
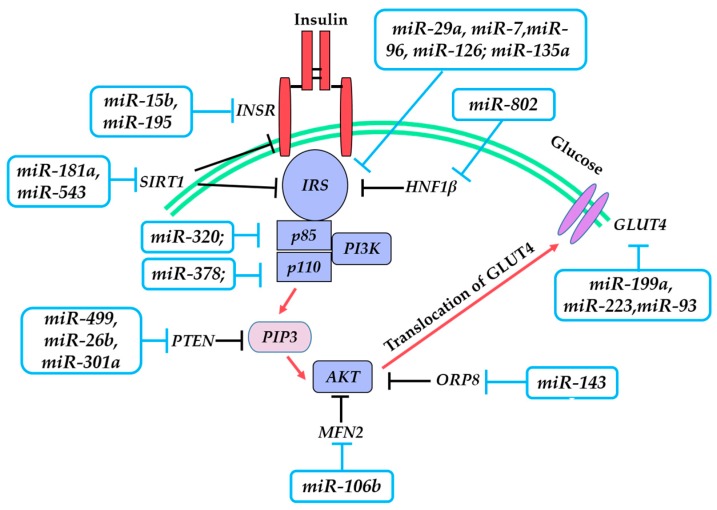
Major protein cascade and miRNAs in insulin signaling pathway. Red arrow—activation; T arrow—direct inhibition (blue); indirect inhibition (black). Abbreviations: *INSR—insulin receptor*; *IRS—insulin receptor substrate*; *PI3K*—*phosphoinositide 3-kinase*; *SIRT1*—*sirtuin 1*; *PTEN*—*phosphatase and tensin homolog*; *PIP3*—*phosphatidylinositol 3,4,5-triphosphate*; *MFN2*—*mitofusin 2*; *AKT*—*AKT serine/threonine kinase*; *ORP8*—*oxysterol binding protein like 8*; *HNF1β*—*hepatocyte nuclear factor 1-β*; *GLUT4*—*glucose transporter 4*.

IR refers to the impaired cellular response to insulin and the inability of normal amounts of insulin to achieve normal glucose homeostasis, which is a hallmark of T2DM. In this process, the insulin signaling pathway plays a central role. It is a highly complicated network that is initiated by insulin binding to the insulin receptor (INSR) on the cell surface, followed by the insulin receptor substrate launching downstream signaling cascades including phosphoinositide 3-kinase (PI3K), AKT serine/threonine kinase (AKT), and glucose transporter 4 (GLUT4). This process is illustrated in Figure 1. A growing body of evidence indicates that IR is associated with defects in insulin signaling. Notably, miRNAs may link insulin signaling and IR.

### 3.1. Insulin Receptor (INSR)

The ligand-receptor interaction is the first step of insulin signaling. Mice lacking the *INSR* gene suffer from hyperglycemia and hyperinsulinemia, and a large number of studies reveal a decrease in *INSR* in T2DM patients [38]. These findings support the importance of *INSR* for maintaining insulin sensitivity. Previous studies also demonstrate that *miR-195* and *miR-15b* are both increased in the livers of obese T2DM model animals, accompanied by the downregulation of *INSR*, and further analysis confirms the direct binding of these miRNAs to the 3′-UTR of *INSR*, resulting in impairment of insulin signaling in hepatocytes [39,40].

### 3.2. Insulin Receptor Substrate 1/2 (IRS-1/2)

Insulin receptor substrate 1 (IRS-1) serves as the key molecule in the insulin signaling pathway in peripheral tissues by transmitting the signals from the INSR to the downstream enzymes. The *IRS-1* expression level is lower in the skeletal muscle of obese-type T2DM mice and humans, indicating that the downregulation of *IRS-1* is associated with IR and T2DM. In saturated fatty acids and high fat diet-induced insulin-resistant L6 myocytes, *miR-29a* expression is increased and represses *IRS-1* expression via the direct targeting of the 3′-UTR of *IRS-1* [41]. Similarly, in another cellular IR model (C2C12 myoblasts pretreated with fatty acids), overexpression of *miR-7* downregulates *IRS-1* expression via a direct interaction through binding to its 3′-UTR [42]. In IR resulting from mitochondrial dysfunction, significant upregulation of *miR-96* is found in SK-Hep1 cells and, consequently, impairs insulin signaling through targeting the 3′-UTR of *IRS-1* [43]. In maternal diet-induced obesity of offspring and IR in later life, *miR-126* plays a negative role via targeting *IRS-1* [44].

Aside from the miRNAs that directly bind to *IRS-1*, a class of miRNAs indirectly inhibits the expression of *IRS-1*. For example, in livers and adipose tissues of diet-induced obesity mice, overexpression of *miR-103/107* negatively regulates insulin signaling by targeting *Cav-1*, a caveolae protein that activates insulin signaling by stabilizing the interaction between caveolae and IRS-1. Thus, enhanced *miR-103/107* levels are concomitant with a decreased stability of *IRS-1* [45].

Insulin receptor substrate 2 (IRS-2) is the alternative substrate of the INSR in *IRS-1*-deficient conditions. In skeletal muscle C2C12 cells, *miR-135a* targets *Irs-2* by binding to its 3′-UTR, and this interaction negatively regulates insulin signaling [46].

### 3.3. Phosphoinositide 3-Kinase (PI3K)/AKT Serine/Threonine Kinase (AKT)

PI3K is an indispensable kinase downstream of IRS-1 in the insulin signaling pathway. In response to the signal from IRS, PI3K is activated and leads to phosphorylation of AKT. PI3K consists of the regulatory subunit P85 and the catalytic subunit P110. The latter exists in two main isoforms in non-leukocytotic cell types, namely, P110α and P110β. Many IR effects are attributed to PI3K. For instance, *miR-378* inhibits insulin signaling by targeting *p110α* in hepatocytes of ob/ob mice [47], and *miR-320* decreases insulin sensitivity in 3T3-L1 adipocytes by targeting the *p85* unit of PI3K [48].

The product of PI3K activation is phosphatidylinositol 3,4,5-triphosphate (PIP3), and PTEN negatively regulates PI3K signaling by dephosphorylating PIP3. Therefore, miRNAs targeting *PTEN* such as *miR-499*, *miR-26b*, and *miR-301a* are beneficial for improving insulin sensitivity [49,50,51].

### 3.4. Glucose Transporter 4 (GLUT4)

Activation of PI3K/AKT induces the translocation of GLUT4 from intracellular vesicles to the plasma membrane. As the last step in the insulin signaling pathway, GLUT4-mediated glucose uptake plays a crucial role in maintaining glucose homeostasis. *GLUT4* can be directly inhibited by *miR-199a* and *miR-93/223* in skeletal muscle and adipose tissue, respectively, which consequently has a negative impact on insulin sensitivity [52,53,54].

In addition to miRNAs regulating key proteins in insulin signaling, they may represent another crucial regulatory layer in regulating insulin sensitivity. *miR-143a*, *miR-802*, and *miR-181a/543* affect hepatic insulin signaling by targeting *Oxysterol binding protein like 8* (*Orp8*), *Hepatocyte nuclear factor 1-β* (*Hnf1β*), and *Sirtuin 1* (*SIRT1*), respectively [55,56,57,58]. *miR-106b* expression closely correlates with skeletal muscle IR by binding to the 3′-UTR of *Mitofusin 2* (*Mfn2*) [59]. Principle miRNAs involved in IR are summarized in Table 2.

## 4. Systematic Analysis of miRNA–mRNA Regulatory Network in Diabetes

It has been shown that a single miRNA is directly responsible for the repression of hundreds of proteins, and a protein coding gene can be modulated by more than one miRNA [60]. Construction of the interaction network of miRNAs and mRNAs will help to elucidate diabetes pathogenesis on a systematic level. To achieve this goal, high-throughput technologies such as sequencing or microarrays are applied, and bioinformatics analysis is used to integrate multiple-omics datasets. Our previous work established the miRNA–mRNA functional network in Goto-Kakizaki (GK) diabetic rats.

Using the pancreatic tissues from GK diabetic rats, we examined the mRNA and miRNA expression profiles by microarray analysis. A total of 19 miRNAs were significantly downregulated or upregulated in pancreas islets of GK diabetic rats compared with normal Wistar rats. By virtue of the existing target prediction databases, target gene candidates were selected for the 19 differentially expressed miRNAs. As mentioned above, miRNAs regulate gene expression through mRNA degradation or translational repression. Thus, we assumed that there would be a negative correlation between miRNA expression and its target mRNA expression level. The predicted target genes were compared with the transcriptome data, and only those miRNA–mRNA pairs displaying opposite expression patterns were further included. In this way, 13 novel miRNAs (including *miR-150*, *miR-497*, and *miR-344-3p*) were found to show differential expression patterns between GK diabetic rats and normal Wistar rats for the first time, providing more new potential targets for research on T2DM. miRNA–mRNA pairs such as *let-7f*-collagen were identified in the study, which may have pivotal functions in pathogenesis and the development of diabetes, meriting further study [61].

Similarly, Tyler et al. use an integrative miRNA–mRNA microarray approach to identify the regulatory network in adipose tissue of insulin sensitive (IS) and IR individuals. Seventeen miRNAs are differentially expressed in the IR vs. IS group, including 16 miRNAs downregulated in IR individuals, such as *miR-30b*, and *miR-145*. Correspondingly, the potential target genes of these miRNAs, i.e., *ADAM22*, *MYO5A*, *LOX* and *GM2A* for *miR-145*; *ADAM22* for *miR-30b*, were upregulated in IR subjects [62].

Along with the high-throughput data generated from sequencing or microarrays, construction of miRNA–mRNA regulatory networks in diabetes will provide new drug or genetic therapeutic candidates of this disease from a systematic and comprehensive view. This will supplement the current knowledge of diabetes in a rapid way, but the identifications from in silico analyses need further validation by molecular studies.

## 5. miRNAs as Type 2 Diabetes (T2DM) Markers and Its Complications

Although the mechanism of T2DM is not definitively clear, and a total cure is still difficult, preventive interventions could be achieved based on early molecular diagnosis. Hence, it is of great significance to discover novel and reliable biomarkers that can predict the onset or progression of T2DM. Circulating miRNAs, as a novel group of miRNAs existing outside cells, have drawn much research interest. The unique expression patterns, stability in circulation, and non-invasiveness of circulating miRNAs make them promising biomarkers for the diagnosis and prognosis of cancers, cardiovascular diseases, and T2DM.

Circulating miRNAs can exist in various types of body fluids, such as serum, plasma, and urine. They are secreted by donor cells via incorporation into vesicles including exosomes, microvesicles, or apoptotic bodies. Increasing evidence has indicated the potential of circulating miRNAs as predicting markers for diabetes and its complications. Zhang et al. previously examined the expression of a panel of plasma miRNAs in three groups of people: normal individuals (fasting glucose, 4.8–5.2 mmol/L), T2DM-susceptible individuals (fasting glucose, 6.1–6.9 mmol/L), and T2DM patients (fasting glucose, ≥7.0 mmol/L). They found that miR-126 is the only miRNA whose expression is significantly reduced in susceptible individuals and T2DM patients compared with normal individuals, suggesting that plasma *miR-126* may serve as a potential marker for the early prediction of susceptible individuals to T2DM [63]. If the *miR-126* expression is found to be lower than 35 (relative quantification unit), the individual is likely to develop T2DM in the following two years [64]. Similarly, by comparing expression profiles of miRNAs in the plasma of patients with prediabetes and newly diagnosed T2DM, Yan et al. demonstrated plasma *miR-1249*, *miR-320b*, and *miR-572* levels as potential biomarkers for early diagnosis of T2DM [65].

One large threat of T2DM for human health is its complications. Several lines of evidence indicate that circulating miRNAs can both indicate the onset of T2DM itself and possess pre-clinical significance for assessment of diabetic complications. For instance, diabetic nephropathy (DN) is one of the most serious complications of diabetes. Recently, a prospective case-control study showed that *miR-21*, *miR-29a/b/c*, and *miR-192* reflect DN pathogenesis and could be of clinical significance to monitor and prevent DN advancement [66]. *miR-21* was shown to suppress *PTEN*, a key modulator in DN [67]; *miR-192* was shown to suppress *ZEB2*, which is responsible for controlling TGF-β-induced extracellular matrix proteins accumulating during DN [67], while *miR-29c* was shown to inhibit *SPRY1*, which involves albuminuria and kidney mesangial matrix accumulation in diabetic mice models [68]. Interestingly, another type of miRNA that may serve as a candidate marker for DN is urinary miRNAs. Aberrant urinary *miR-320c*, isolated from urinary exosomes, may impact the TGF-β signaling pathway via targeting *THBS1* and could be used as a novel candidate marker for disease progression in DN [69].

Coronary artery disease (CAD) is another complication of T2DM, and circulating *miR-126* displays differential expression patterns in the plasma of T2DM patients, T2DM patients with CAD, and healthy subjects, suggesting the potential of circulating *miR-126* as a biomarker for diabetic CAD [70].

## 6. Concluding Remarks

Since their first discovery in *Caenorhabditis elegans* in 1993, miRNAs have attracted increasing attention. As a class of short non-coding RNAs, miRNA shed new light into the understanding of the genome. Considerable progress has already been achieved in the research of the links between regulatory miRNAs and diseases such as cancers, neurological diseases, and diabetes. miRNAs play pivotal roles in the onset and development of diabetes by affecting pancreatic β-cell function, IR, or both. Research into miRNA functions systematically provides us with valuable information to delineate the complex regulatory networks between miRNAs and mRNAs. Moreover, studies in recent years confirm that miRNA exists both inside and outside cells. The exciting emergence of circulating miRNA implicates them as an attractive choice for non-invasive biomarkers for the diagnosis and prognosis of T2DM. As potential disease biomarkers, miRNAs exhibit convincing advantages: sensitivity, stability, non-invasiveness, and reproducibility. A number of miRNA candidate signatures have appeared, and clinical trials are underway to validate their effectiveness.

## Figures and Tables

**Table 1:** Principle microRNAs (miRNAs) involved in pancreatic β-cell function.

Cell Processes	miRNA	Putative Targets	Cells or Tissues Studied	Species	References
β-cell survival/apoptosis	*miR-577*	*Fgf-21*	INS-1 cells	rat	[11]
	*miR-200a/b/c*	-	islets of diabetic mice	mouse	[12]
	*miR-34a*	*Bcl-2*	MIN-6 cells	mouse	[13]
β-cell proliferation	*miR-375*	*Cadm1*	islets of *miR-375* KO mice; INS-1E cells	mouse; rat	[14,15]
	*miR-181a*	*Pdgfrα*	3- and 12-month-old rat islets	rat	[16]
	*miR-17*	*Menin*	MIN-6 cells	mouse	[17]
	*miR-24*	*Hnf1α*; *Neurod1*	islets of obese mice	mouse	[19]
	*miR-29a*	-	INS-1E cells	rat	[20]
β-cell differentiation	*miR-375*	*HNF1β*	hPSCs differentiated IPCs	human	[21]
	*miR-7*	*PAX6*	hPSCs differentiated IPCs	human	[21]
	*miR-34a*	-	hPSCs differentiated IPCs	human	[21]
	*miR-146a*	-	hPSCs differentiated IPCs	human	[21]
	*miR-30d*	*RFX6*	hPSCs differentiated IPCs	human	[25]
	*let-7e*	*RFX6*	hPSCs differentiated IPCs	human	[25]
	*miR-21*	*SOX6*; *RBJ*	pancreatic progenitor cells	human	[26]
	*miR-9*	-	human fetal islets	human	[27]
	*miR-376*	-	human fetal islets	human	[27]
Insulin secretion	*miR-375*	*Mtpn*	MIN-6 cells	mouse	[28]
	*miR-184*	*Ago 2*	MIN-6 cells	mouse	[29]
	*miR-7a*	-	β-cells of diabetic mice	mouse	[30]
	*miR-29a*	*Stx-1a*; *Mct1*	rat islets; mouse islets	rat; mouse	[31,32]
	*miR-187*	*HIPK3*	human islets from T2DM patients	human	[33]
	*miR-30a*	β*2*; *NeuroD1*	rat islets and INS-1 cells	rat	[34]
	*miR-124*	*Rab27a*; *Foxa2*	human islets; MIN-6 cells	human; mouse	[35,36]
	*miR-33*	*Abca1*	mouse islets; MIN-6 cells	mouse	[37]

**Table 2:** Principle miRNAs involved in insulin resistance (IR).

Cell Processes	miRNA	Putative Targets	Cells or Tissue Studied	Species	References
IR	*miR-15b*	*INSR*	hepatocytes of diabetic mice	mouse	[39]
	*miR-195*		HepG2 cells	human	[40]
	*miR-29a*	*IRS-1*	L6 cells	rat	[41]
	*miR-7*		C2C12 cells	mouse	[42]
	*miR-96*		SK-Hep1 cells	human	[43]
	*miR-126*		adipose tissue	human	[44]
	*miR-135a*	*Irs-2*	C2C12 cells	mouse	[46]
	*miR-378*	*PI3K*	hepatocytes of miR-378/378 * knockout mice	mouse	[47]
	*miR-320*		3T3-L1 cells	mouse	[48]
	*miR-199a*	*GLUT4*	L6 cells	rat	[52]
	*miR-223*		human differentiated adipocytes	human	[53]
	*miR-93*		subcutaneous adipose tissue	human	[54]
	*miR-499*	*PTEN*	NCTC 1469 cells; livers of db/db mice	mouse	[49]
	*miR-26b*		human insulin-resistant viceral adipocytes	human	[50]
	*miR-301a*		NCTC 1469 cells	mouse	[51]
	*miR-143*	*Orp8*	HL-1 cells	mouse	[55]
			livers of obese mice	mouse	[56]
	*miR-181a*	*SIRT1*	HepG2 cells	human	[57]
	*miR-543*		HepG2 cells	human	[57]
	*miR-29*	*Pparδ*	C2C12 cells	mouse	[53]
	*miR-103/107*	*Cav-1*	livers and adipose tissues of diet induced obesity mice	mouse	[45]
	*miR-802*	*Hnf1β*	Hepa1-6 cells	mouse	[58]
	*miR-106b*	*Mfn2*	C2C12 cells	mouse	[59]

IR—Insulin resistance; miR-378/378 *—*miR-378* and miR-378 * are two mature microRNAs originate from opposite arms of the same pre-miRNA. An asterisk following the name indicates the mature species found at low levels in cells.

## Appendix A

**Table A1:** Gene symbol abbreviations and full names.

Gene Symbol Abbreviations	Gene’s Full Names
*Bcl-2*	*B-cell lymphoma-2*
*Trp53*	*transformation related protein 53*
*Bax*	*BCL2 associated X, apoptosis regulator*
*Cadm1*	*cell adhesion molecule 1*
*Hnf1α*	*hepatocyte nuclear factor 1 homeobox A*
*Neurod1*	*neurogenic differentiation 1*
*HNF1β*	*hepatocyte nuclear factor 1 homeobox β*
*PAX6*	*paired box 6*
*Stx-1*	*syntaxin 1*
*Rab27a*	*Ras-related protein Rab-27A*
*Foxa2*	*forkhead box protein A2*
*Fgf21*	*fibroblast growth factor 21*
*Pdgfrα*	*platelet-derived growth factor receptor, α polypeptide*
*RFX6*	*regulatory factor X6*
*SOX6*	*sex determining region Y-box 6*
*Mtpn*	*myotrophin*
*Ago2*	*argonaute 2*
*Mct1*	*monocarboxylate transporter 1*
*HIPK3*	*homeodomain interacting protein kinase 3*
*Abca1*	*ATP binding cassette subfamily A member 1*
*INSR*	*insulin receptor*
*IRS-1*	*insulin receptor substrate-1*
*IRS-2*	*insulin receptor substrate-2*
*PI3K*	*phosphatidylinositol 3-kinase*
*GLUT4*	*glucose transporter member 4*
*PTEN*	*phosphatase and tensin homolog*
*Orp8*	*oxysterol binding protein like 8*
*SIRT-1*	*sirtuin 1*
*Mfn2*	*mitofusin 2*
*ADAM22*	*ADAM metallopeptidase domain 22*
*MYO5A*	*myosin VA*
*LOX*	*lysyl oxidase*
*GM2A*	*GM2 ganglioside activator*
*ZEB2*	*zinc finger E-box binding homeobox 2*
*TGF-β*	*transforming growth factor β*
*SPRY1*	*sprouty homolog 1*
*THBS-1*	*thrombospondin 1*
